# Target tissue insulin resistance in early and late onset of overweight or obesity

**DOI:** 10.1038/s41366-026-02146-8

**Published:** 2026-07-02

**Authors:** Alexander Zadruzny, Peter Arner, Daniel P. Andersson

**Affiliations:** 1 Department of Medicine-H7 at Karolinska Institutet, Karolinska University Hospital Huddinge, Stockholm, Sweden; 2 Department of Endocrinology, Karolinska University Hospital Huddinge, Stockholm, Sweden

**Keywords:** Obesity, Translational research

## Abstract

**Background/Objective:**

Overweight and obesity are associated with insulin resistance. However, the importance of the time of onset of excess body fat is unknown and was presently examined.

**Subjects/Methods:**

We included 339 adult participants having information about their body mass index (BMI) at 18 years of age. Insulin action was determined as homeostasis model assessment (HOMA-IR) reflecting liver, hyperinsulinemic euglycemic clamp reflecting skeletal muscle, Adipo-IR reflecting adipose tissue in vivo, and insulin action on lipogeneses reflecting fat cells. The subjects were divided into never having overweight/obesity with BMI always <25 kg/m^2^ (NO), having BMI ≥ 25 kg/m^2^ already at 18 years of age (EO), and late onset of overweight when BMI was ≥25 kg/m^2^ only at current examination (LO). The groups were compared by unpaired *t*-test and single and multiple regression analysis (the latter to study the influence of other factors than insulin action).

**Results:**

EO had 5 kg/m^2^ higher BMI and was 10 years younger than LO at examination (*p* < 0.0001). EO was more insulin resistant than LO for both HOMA-IR and Adipo-IR, but not clamp (*p* = 0.01, 0.02, and 0.11, respectively). However, when the different measures of insulin resistance were corrected for current BMI or age there were no significant differences between EO and LO for any of the measures of insulin action (*p* ≥ 0.08). Furthermore, in all subjects current BMI (*p* < 0.0001) but not BMI when 18 years old (*p* ≥ 0.13) correlated with different insulin resistance measures.

**Conclusion:**

When current BMI or age is considered, there is no difference between early or late onset of overweight/obesity for the level of insulin resistance in the different target tissues of the hormone.

## Introduction

Insulin resistance (IR) is a major feature of obesity and an important contributor to the development of type 2 diabetes [1]. IR is also present with moderate excess of body fat, as exemplified [2, 3]. In addition, IR is present in adolescents or children with obesity as reviewed [4, 5].

An unanswered question is whether IR differs between early and late onset of overweight/obesity. This is of considerable clinical relevance because the risk of developing type 2 diabetes is particularly high when there is an early onset of obesity [6].

To shed new light on the importance of the time of onset of overweight/obesity for IR we investigated the major target tissues of the hormone (skeletal muscle, liver, and adipose tissue) and fat cells in adult subjects having information on their body mass index (BMI) at 18 years of age. A BMI value of 25 kg/m^2^ as cut-off at 18 years and at the current examination was used to divide the participants into those never having overweight or obesity (NO), having the conditions already at an early age (EO) or having developed excess body fat during adulthood (LO).

## Research design and methods

### Subjects

From 1996 to 2022 we included 339 adults (86 men) who gave information about BMI at 18 years of age and were willing to undergo the suggested examinations of IR. Sex was determined at birth and confirmed at examination. All lived in the Stockholm, Sweden, area and were healthy according to self-report. They were contacted by local advertisement. A never-used exclusion criterion was ongoing severe acute or chronic disorders. About 5% were of non-European origin. Subjects were divided into never overweight (NO, BMI always <25 kg/m^2^), early onset of overweight (EO, BMI ≥ 25 kg/m^2^ from age 18), and late onset of overweight (LO, BMI ≥ 25 kg/m^2^ only at current examination). According to the mentioned classification of onset of overweight or obesity the numbers of NO, LO and EO participants were 61, 178, and 100, respectively. Four subjects had a BMI > 25 kg/m^2^ at 18 years of age but with normal BMI values (<25 kg/m^2^) at the current examination. They were excluded.

We recently demonstrated that self-reported BMI is a reasonably valid measure of true BMI [7]. Several of the presently investigated subjects also participated in the previous study, which dealt with another topic, namely fat cell size and fat cell number among subjects with early or late onset of overweight/obesity [7]. The subjects have also been included in other types of studies as exemplified [8]. All previous and present studies have been approved by the local committee on ethics in Stockholm. In the most recent permits from 31 January 2018 (register number 809-31) and 18 May 2025 (register number 2025-03186-02), we were allowed to retrospectively analyze all previously recorded results as was done presently. The study was performed in accordance with the Declaration of Helsinki. The study was explained in detail to each participant, and his/her informed written consent was obtained.

### Clinical and adipose examinations

The participants came to the laboratory in the morning after an overnight fast. According to self-report they were body weight stable for at least three months (<2 kg change). Blood pressure was determined, anthropometric and routine clinical chemistry measures were performed and followed by an abdominal subcutaneous needle biopsy, using prilocaine hydrochloride as an anesthetic agent for collection of adipose samples as described [3, 7, 8]. We determined four different aspects of IR. Because of the availability of staff and willingness to participate in different types of investigations it was not possible to perform all IR measures in all participants.

In a subset of subjects, a hyperinsulinemic euglycemic clamp was performed as described [9]. Briefly, after a bolus dose of insulin (1.6 U/m^2^ body surface area), insulin was infused at a rate of 120 mU/m^2^ body surface area/minute for 2 h. Euglycemia was maintained by a variable intravenous glucose infusion (200 mg/ml). Glucose uptake during the last hour of clamp was determined (M-value). Under these conditions with high circulating insulin levels hepatic glucose release is shut off so that glucose uptake mainly represents hormone action on skeletal muscle [10]. Fasting plasma glucose and serum insulin values were used to calculate HOMA-IR as described [11]. These values are suggested to represent IR in the liver [12]. Fasting circulating serum values of fatty acids and insulin were used to construct Adipo-IR values as described [8, 13], which are shown to correlate reasonably well with the antilipolytic sensitivity of insulin in adipose tissue [14].

When enough adipose tissue was available, insulin-stimulated fat cell lipogenesis was determined as described [8]. Briefly, isolated fat cells were prepared and incubated in duplicate without or with human insulin at different concentrations for 2 h at 37 °C in a buffer containing tritiated glucose. The incorporation of radioactive glucose into the total lipids of fat cells was used as index of lipogenesis. The ratio of lipogenesis at maximum effective insulin concentration divided with lipogenesis without insulin was calculated and represented the level of IR in fat cells.

### Statistical methods

Analyses were performed in JMP Version 16.1.0 (SAS, Institute Inc., Buckinghamshire, UK) and Figure in GraphPad Prism Version 10.0.2 (GraphPad Software, Boston, USA). Values are mean and (range) in tables and box plots in figures. Values for HOMA-IR, Adipo-IR and lipogenesis were 10log transformed for purpose of normalization. The first comparison was of NO, LO, and EO using analysis of variance. The second comparison was of LO and EO using unpaired *t*-test followed when stated by analysis of covariance. The third comparison used single or multiple regression with LO/EO as primary independent factor versus different measures of insulin. The latter analysis models contained the following co-factors, which were considered important for insulin action besides early/late overweight/obesity [1, 15–18]: age, sex, current BMI, physical activity and waist-to-hip ratio. These cofactors were analyzed together with the onset of overweight/obesity one at a time in different models of analysis of covariance. We preferred to analyze each cofactor separately because they may interact with each other as regards the influence on IR [1, 15–18]. All tests were two-sided. A *p*-value of <0.05 was considered being statistically significant. When individuals were divided into NO, LO, and EO based on the BMI at 18 years of age, current BMI (i.e. not BMI at 18 years) was used in the analyses, except when looking at the whole group and the influence of current BMI or BMI at age 18 years of age.

Prior to finalizing the inclusion of participants, we made a statistical power calculation. For this purpose, we used our previously published study group on sex differences in IR [17] to determine mean ± SD for 10log HOMA-IR in subjects with overweight or obesity. The values were 0.55 ± 0.36. Using these numbers we could determine a 25% difference in HOMA-IR between two groups with equal size of 100 subjects at *p* = 0.05 with 80% power. This indicated that we had sufficiently large LO and EO groups to detect rather small differences in the levels of insulin resistance for most of the insulin action measures.

## Results

Table 1 shows the clinical characteristics of the three subgroups. Those with overweight or obesity showed the expected differences from those who were of normal weight. There were also some differences between EO and LO. The most prominent ones were that EO was 10 years younger and had about 5 kg/m^2^ higher current BMI than LO. The rate of increase in BMI from 18 years and onwards was three times more rapid in EO than LO. EO were also more sedentary than LO. In addition, EO participants were investigated on average one year earlier than LO and NO participants (2008 versus 2009).

**Table 1:** Clinical data for subjects always being lean (NO), having overweight or obesity at 18 years of age and at current examination (EO) and subjects having overweight or obesity only at current examination (LO).

Phenotype	Group			*p*-value	
	NO (*n* = 61)	LO (*n* = 178)	EO (*n* = 100)	Three groups	EO vs LO
Age (years)	48 (19–78)	50 (24–72)	40 (18–79)	<0.0001	<0.0001
Sex (males/females)	16/45	49/129	21/79	0.48	0.25
Body mass index (kg/m^2^) at present	22.7 (17.7–24.9)	33.8 (25.0–50.0)	39.0 (25.0–57.0)	<0.0001	<0.0001
Body mass index (kg/m^2^) at 18 years	20.5 (15.7–24.7)	21.5 (16.2–25.0)	29.9 (15.1–45.0)	<0.0001	<0.0001
Body mass index at present – at 18 years of age (kg/m^2^)	2.2 (-2.1–9.1)	12.3 (1.0–27.1)	9.2 (-8.2–26.5)	<0.0001	<0.0001
Rate of changes in body mass index over time (kg/m^2^/year)	0.0088 (-0.089–0.56)	0.47 (0.04–3.53)	0.61 (-0.054–3.45)	<0.0001	0.01
Waist to hip (ratio)	0.86 (0.73–0.99)	0.97 (0.78–1.15)	0.97 (0.80–1.14)	<0.0001	0.68
S-insulin (mU/l)	5 (2–14)	11 (2–41)	15 (3–48)	<0.0001	0.003
P-glucose (mmol/l)	5.3 (4.4–8.7)	5.7 (4.3–12.2)	5.6 (4.3–14.2)	0.06	0.36
P-triglycerides (mmol/l)	0.8 (0.3–2.5)	1.3 (0.0–6.9)	1.3 (0.4–4.7)	<0.0001	0.65
P-total cholesterol (mmol/l)	4.6 (2.7–6.5)	4.9 (2.7–9.4)	4.6 (2.5–7.8)	0.006	0.007
P-HDL cholesterol (mmol/l)	1.57 (0.60–2.80)	1.30 (0.80–2.20)	1.18 (0.60–3.30)	<0.0001	0.001
S-fatty acids (mmol/l)	0.59 (0.14–1.67)	0.67 (0.07–1.40)	0.66 (0.20–1.97)	0.13	0.90
Systolic blood pressure (mm Hg)	122 (84–156)	134 (86-182)	134 (96–179)	<0.0001	0.92
Diastolic blood pressure (mmol/l)	74 (55–103)	81 (45-125)	80 (54–104)	<0.0001	0.22
Physical activity (sedentary/active)	2/58	38/139	32/67	<0.0001	0.046
Time of investigation (calendar year)	2009 (1992–2016)	2009 (1992–2016)	2008 (2002–2015)	0.022	0.04

Values are mean and (range). All three groups were compared with analysis of variance and EO was compared with LO using unpaired *t*-test. For sex and physical activity distribution results were compared by chi-square. Rate of changes in body mass index over time was calculated as current value minus value at 18 years divided by current age minus 18. Number of subjects = *n*.

**Fig. 1:**
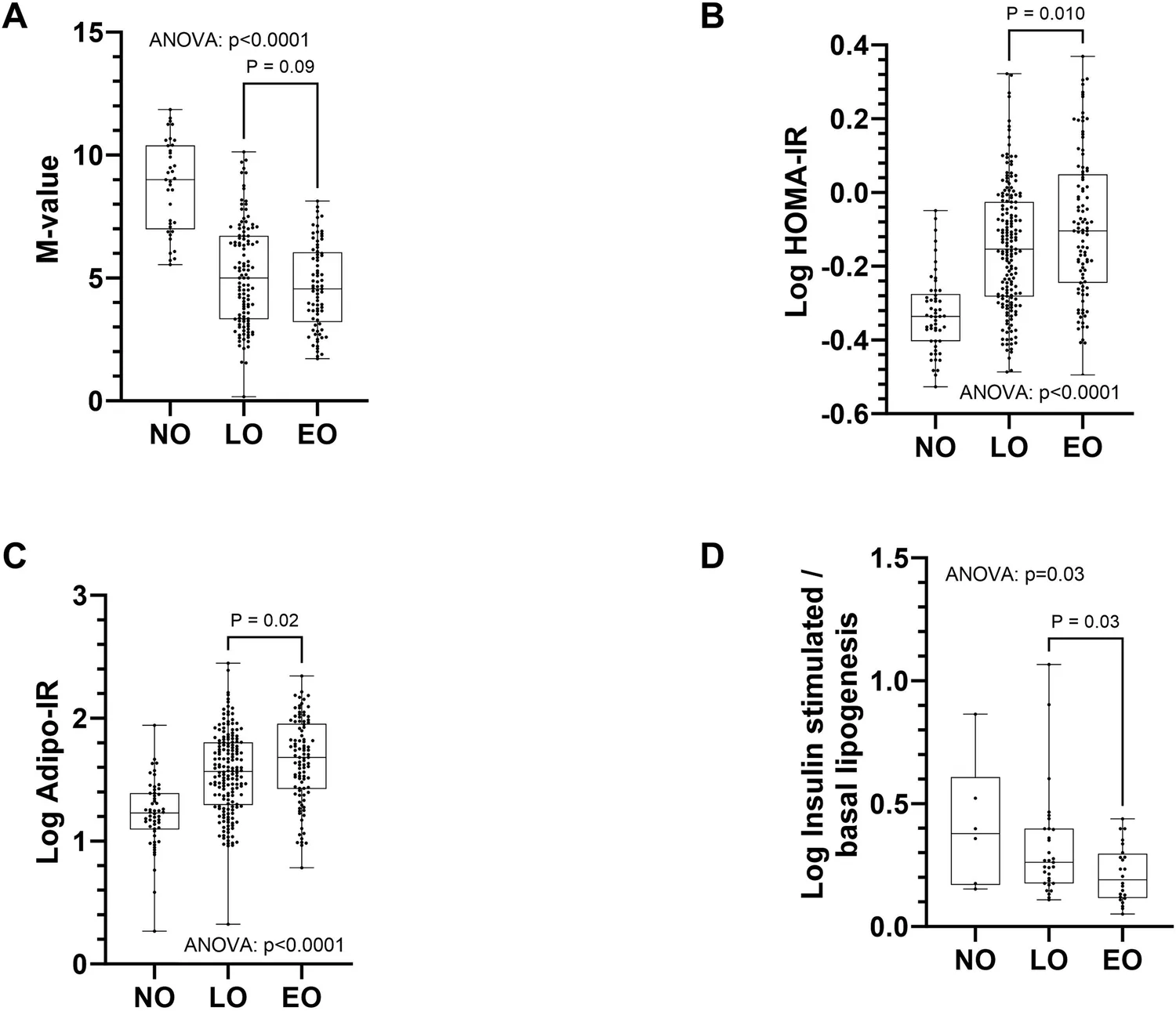
Insulin effects in subjects always being lean (NO), having overweight or obesity at 18 years of age and at present examination (EO), or having overweight or obesity only at present examination (LO). M-value is glucose disposal rate during euglycemic hyperinsulinemic clamp (mg glucose/kg body weight/minute). HOMA-IR is 10 log value for serum insulin times plasma glucose divided by 22.5. Adipo-IR is 10 log value for serum insulin times serum fatty acids. Insulin/basal lipogenesis is 10 log value for glucose incorporation into isolated subcutaneous fat cells in the presence of maximum effective insulin concentration divided by the values when no insulin is present (basal). Numbers of subjects in (**A**) are for NO 37, 116 for EO, and 77 for LO. Corresponding numbers are 54, 174, and 99 for (**B**), 54, 173, and 98 for (**C**) and 6, 31, and 24 for (**D**). The three groups were compared by analysis of variance. LO and EO were compared by unpaired *t*-test.

The findings with the four measures of IR (clamp, HOMA-IR, Adipose-IR and lipogenesis) are depicted in Fig. 1. For all measures EO and LO were more insulin resistant than NO. The figure also shows that EO compared to LO were more insulin resistant regarding adipose tissue insulin resistance (Adipo-IR and ratio of insulin stimulated/basal lipogenesis), as well as hepatic insulin resistance (HOMA-IR) but not whole body insulin resistance (clamp).

Sex, body fat distribution, age, BMI and physical activity may influence IR. We investigated whether these factors were important or not for the differences between EO and LO as regards IR by analysis of covariance (Table 2). Five different models of multiple regression were used. The number of subjects undergoing clamp or measuring lipogenesis was small. Therefore, the analyses were focused on Adipo-IR and HOMA-IR. BMI, waist-to-hip ratio, and physical activity significantly influenced both HOMA-IR and Adipo-IR, whereas only age and sex influenced Adipo-IR in this way. The differences between LO and EO for IR values disappeared as regards statistical significance when considered together with current BMI or age, but were statistically maintained when IR was examined together with waist-to-hip ratio or sex. For physical activity only the difference between LO and EO was statistically maintained for HOMA-IR. Thus, the difference in IR between LO and EO was not independent. Most evident was the influence of age and current BMI reducing possible differences in IR between the two groups of overweight/obesity to statistically non-significant levels.

**Table 2:** Influence of cofactors on the relationship between late (LO) or early (EO) onset of overweight/obesity versus insulin action parameters (HOMA-IR or Adipo-IR) using different models of multiple regression.

Model	HOMA-IR (10 log units)				Adipo-IR (10 log units)			
	Estimate	*t*-ratio	Prob > |*t*|	95% CI	Estimate	*t*-ratio	Prob > |*t*|	95% CI
Model 1								
LO versus EO	0.006	0.52	0.60	−0.015–0.026	0.035	1.93	0.05	−0.001–0.070
Body mass index (kg/m^2^) at present	0.014	9.09	<0.0001	0.011–0.017	0.032	12.76	<0.0001	0.027–0.037
Model 2								
LO versus EO	−0.022	−1.75	0.08	−0.056–0.003	−0.024	−1.07	0.29	−0.069–0.021
Age (years)	−0.002	−1.63	0.10	−0.004–0.0003	−0.005	−2.70	0.007	−0.009–0.001
Model 3								
LO versus EO	−0.028	−2.74	0.007	−0.048–0.008	−0.048	−2.40	0.017	−0.088–0.009
Waist to hip (ratio)	1.03	7.46	<0.0001	0.76–1.30	1.56	5.82	<0.0001	1.03–2.09
Model 4								
LO versus EO	−0.029	−2.56	0.01	−0.051–0.007	−0.04	−2.06	0.04	−0.083–0.002
Sex (male or female)	0.005	0.43	0.67	−0.19–0.030	0.10	4.62	<0.0001	0.060–0.149
Model 5								
LO versus EO	−0.025	−2.277	0.02	−0.047–0.003	−0.036	−1.77	0.08	−0.077–0.004
Sedentary or physically active	−0.044	−3.56	0.0004	−0.068–0.020	−0.11	−5.00	<0.0001	−0.158–0.069

LO/EO was investigated together with body mass index at current investigation (Model 1), age (Model 2), waist-to-hip ratio (Model 3), sex (Model 4) or physical activity (Model 5). The number of subjects was between 271 and 274 in each analysis.

We further investigated the importance of BMI for IR by analyzing all individuals together (no table or figure). For this analysis, the subjects with lipogenesis were too few for a valid examination. When current BMI or BMI at 18 years of age were investigated alone both BMI measures correlated significantly with M-values for clamp (*r*^2^ = 0.52 : *p* < 0.0001 and *r*^2^ = 0.07 : *p* < 0.0001 respectively) and with values for HOMA-IR (*r*^2^ = 0.36 : *p* < 0.0001 and *r*^2^ = 0.07 : *p* < 0.0001 respectively) or Adipo-IR (*r*^2^ = 0.43 : *p* < 0.0001 and *r*^2^ = 0.07 : *p* < 0.0001 respectively) but, only current BMI showed a strong correlation as judged by the *r*^2^-values. Furthermore, when the two BMI measures were examined together only BMI at present was significantly correlated with the different measures of IR (*p* < 0.0001) (Table 3). Thus, although overweight/obesity was present for longer time in EO than LO only current BMI was of importance for the level of IR.

**Table 3:** Influence of body mass index (BMI) at 18 years of age and currently on insulin action parameters.

Independent variables	M-value (glucose disposal in mg/kg body weight/minutes) (*n* = 233)				Log 10 HOMA-IR (serum insulin times plasma glucose divided by 22.5) (*n* = 331)				Log 10 Adipo-IR (serum insulin times serum fatty acids) (*n* = 329)			
	Estimate	*t*-ratio	Prob > |*t*|	95% CI	Estimate	*t*-ratio	Prob > |*t*|	95% CI	Estimate	*p*-value	Prob > |*t*|	95% CI
BMI at 18 years	0.019	0.82	0.41	−0.027–0.065	0.0008	−0.41	0.69	−0.004–0.003	−0.005	−1.52	0.13	−0.012–0.002
BMI currently	−0.23	−14.96	<0.0001	−0.25–0.20	0.014	12.2	<0.0001	0.012–0.017	0.031	14.64	<0.0001	0.027–0.035

The contribution of the two BMI measures to variations in each of the insulin action parameters was investigated by multiple regression. Adjusted *r*-square values for the whole model were 0.53, 0.36, and 0.43 for M-value, HOMA-IR and Adipo-IR, respectively.

See Methods for further details.

## Discussion

This study presents new information on the nature of IR in overweight or obesity states. When confounding factors are considered there is no or little importance of time of onset of excess body fat for the levels of IR in different hormone target tissues.

Irrespective of duration of excess body fat LO and EO showed the characteristic adverse phenotypes compared with NO. There were some minor differences in clinical profile between LO and EO except for that EO was much younger and heavier than LO. It is possible that the latter differences are influenced by the mode of recruitment. We made no attempts to stratify clinical data except regarding BMI values at 18 years and current age. It is apparent, though, from results in Table 1 that EO had a more rapid increase in BMI than LO over time. Several previous studies have demonstrated higher current BMI in EO than LO [7, 19, 20]. It is therefore likely that people with early onset of excess body fat are more prone to increase their BMI over time than those with a late onset of overweight/obesity.

When confounding factors were not considered, EO was more resistant to insulin than LO in all examined tissues except skeletal muscle, as judged by the M-values and values for HOMA-IR, Adipose-IR, or adipocyte lipogenesis. On the other hand, these differences in IR were probably more apparent than real. Age and BMI per se had a strong influence on IR in the various tissues. When these factors were considered in the analysis there was no statistically significant difference between EO and LO as regards the various IR measures. In other words, the time of onset of excess body fat is just a dependent factor for the level of IR because ageing and BMI have strong impact by themselves on IR. For BMI it appears from the analysis in Table 3 that current values are more important for IR than the BMI at adolescent age. This has clinical implications. Although IR in young people with overweight or obesity should not be neglected it might be important for the health status later in life to encourage the young ones not to increase their BMI further when growing up. Clearly, this idea must be validated by direct prospective investigations.

There are some limitations to consider with the present study. Relying on a single self-reported data point to define the onset of overweight/obesity without access to objectively verified measurements constitutes a methodological limitation that is difficult to avoid. This approach may introduce possible confounding, as subjects are categorized without precise information on the onset or duration of overweight/obesity. One limitation is that for individuals with late-onset obesity, information on duration is unavailable. Ideally, potentially in a future study, repeated and professionally obtained anthropometric measurements from infancy through adulthood would be available. Correa-Burrows et al. [21] attempted a similar approach by following ~1000 subjects with regular documented weight measurements. However, even their study lacked BMI data during critical life periods when the risk of developing overweight/obesity is particularly high. Conducting the present study under ideal conditions would therefore require substantial long-term resources to follow a sufficiently large cohort over several decades, estimated at no fewer than 2000 additional subjects based on BMI development over time in the Swedish population. Nevertheless, simplified categorizations of onset of overweight/obesity, like ours, have been widely used in previous research, including Wrzosek et al. [19] and Gourgari et al. [22]. This indicates that such an approach is accepted within the field and is appropriate for addressing our research question. On average the EO group was investigated one year later (2009) than the other two groups (2008). It is unlikely that this small difference in the year of examination has influenced the outcome in a meaningful way.

Furthermore, the study was not population-based. However, subjects who are willing to participate in extensive clamp or invasive (fat biopsy) studies may not be representative for the population at large. It is, on the other hand, unlikely that a selection bias has strongly influenced our results. We investigated a large group of people and used several independent measures of IR, which all gave similar findings. We predominantly investigated participants of European origin, and ethnic variations in IR are reported as reviewed [23]. The number of men was smaller than that for women. Unfortunately, the material was too small for a subdivision according to sex. However, the latter factor is probably of minor importance for the present results because they were independent of sex according to the analysis of covariance in Table 2. Finally, additional elements of importance for IR in overweight/obesity not examined herein such as diet, inflammation, neuronal factors and ectopic fat [24] could have specific impacts linked to onset of excess body fat.

There are also some advantages with the investigation. The number of participants was large enough to detect small differences in IR measures between subgroups. The same group of technical staff was used throughout the study. We simultaneously examined several IR measures including lipogenesis in adipocytes allowing a separation of IR differences between specific target tissues/cells for insulin action

In summary, IR is found in all major target tissues for the hormone when overweight or obesity is present. However, the level of IR is not dependent on the time of onset of excess body fat when current age or BMI is considered.

## Data Availability

All presented data are available on reasonable requests. Requests should be addressed to DPA or PA.
